# Myoblast Myogenic Differentiation but Not Fusion Process Is Inhibited via MyoD Tetraplex Interaction

**DOI:** 10.1155/2018/7640272

**Published:** 2018-05-07

**Authors:** Stefano Testa, Pietro D'Addabbo, Ersilia Fornetti, Roberta Belli, Claudia Fuoco, Sergio Bernardini, Stefano Cannata, Domenico Frezza, Cesare Gargioli

**Affiliations:** ^1^Department of Biology, University of Rome Tor Vergata, Rome, Italy; ^2^Department of Biology, University of Bari, Bari, Italy

## Abstract

The presence of tetraplex structures in the promoter region of the myogenic differentiation 1 gene (MyoD1) was investigated with a specific tetraplex-binding porphyrin (TMPyP4), to test its influence on the expression of MyoD1 itself and downstream-regulated genes during myogenic differentiation. TMPyP4-exposed C2C12 myoblasts, blocking MyoD1 transcription, proliferated reaching confluence and fused forming elongated structures, resembling myotubes, devoid of myosin heavy chain 3 (MHC) expression. Besides lack of MHC, upon MyoD1 inhibition, other myogenic gene expressions were also affected in treated cells, while untreated control cell culture showed normal myotube formation expressing MyoD1, Myog, MRF4, Myf5, and MHC. Unexpectedly, the myomaker (Mymk) gene expression was not affected upon TMPyP4 exposure during C2C12 myogenic differentiation. At the genomic level, the bioinformatic comparison of putative tetraplex sites found that three tetraplexes in MyoD1 and Myog are highly conserved in mammals, while Mymk and MHC did not show any conserved tetraplexes in the analysed regions. Thus, here, we report for the first time that the inhibition of the MyoD1 promoter function, stabilizing the tetraplex region, affects downstream myogenic genes by blocking their expression, while leaving the expression of Mymk unaltered. These results reveal the existence of two distinct pathways: one leading to cell fusion and one guaranteeing correct myotube differentiation.

## 1. Introduction

The G-quartets giving rise to tetraplex structures, also called G-quadruplex, were first observed in telomeric DNA sequences [1]. Since then, putative sites of tetraplex structures have been described in other relevant regions of the genome, for example, in promoter/enhancer regions [2–4]. It was also demonstrated that these regulatory regions can assume different three-dimensional (3D) shapes, depending on the internal nucleotide sequence as well as on other factors, like DNA-binding proteins and epigenetic changes [5–7]. The presence of tetraplex sequences was found to have either an inhibitory or enhancing effect on the gene transcription [8]. Moreover, the position of the tetraplex on the template strand or on the opposite DNA strand can induce one or the other function [9, 10]. Tetraplexes have been identified in various portions of transcribed RNAs, for example, in both the 5′ and 3′UTR, mainly with an inhibitory function [11–13].

Small molecules that stabilize one of the possible 3D shapes of the G-quartets have been hypothesized as tools for antineoplastic function, for example, to block promoters containing tetraplex consensus [14–16]. The oncogenes c-myc, bcl2, and kRAS, the dystrophy gene DMPK, the transcription factor Oct4, and the topoisomerase-1 are all putative targets of those tetraplex-interacting molecules as part of potential clinical care [17–20]. TMPyP4 is one of those molecules, as it is a cationic porphyrin that is known mainly as a DNA tetraplex stabilizer, which is largely used for *in vitro* analysis [21].

The MyoD1 promoter has been studied for the presence of tetraplexes, and putative function of the detected sites has also been investigated for engineered constructs with reporter genes [22, 23]. Since tetraplexes are present in the MyoD1 promoter, in the present study, we used the mouse myoblast cell line C2C12 to investigate the effect of TMPyP4, stabilizing tetraplex sites, on the myogenic differentiation [9, 24]. We used the molecule at a nontoxic concentration and observed the effect on a set of genes known to be involved in muscle differentiation [25, 26]: myogenic differentiation 1 (MyoD1), myogenin (Myog), myogenic factor 5 (Myf5), myogenic regulatory factor 4 (MRF4), myosin heavy chain 3 (MHC), and myomaker (Mymk). The obtained results revealed that inhibiting MyoD1 mRNA transcription appears to involve Myog, Myf5, MRF4, and MHC, but not the fusion gene myomaker.

It is well documented that activation of the myogenic program depends on the MyoD1 expression which induces transcription of downstream target genes involved in the myogenic differentiation [27]. Since the promoters of these genes are highly conserved in animal genomes, we hypothesized that tetraplex DNA positions might follow the same rule if functional constraints were playing their antimutational action. Evolutionary pressure brings to conservation also cis-acting genomic DNA structures like palindromic sequences and G-quartets that form tetraplex *in vitro*, as demonstrated for those in the regulatory region of the immunoglobulin heavy-chain constant genes [28]. We analysed the genomic conservation of cis-acting regions with transcription-control functions, as a tool to predict the possible relevance of the DNA features of our interest. Here, we report the identification through conservation of tetraplex sites and a possible association of these structures to the differentiation of myogenic cells. This is evidenced by the *in vitro* activity of a tetraplex-binding porphyrin that inhibits the MyoD1 activity and the cascade of myogenic differentiation in the C2C12 cell line.

## 2. Material and Methods

### 2.1. Cell Culture

Murine myoblast C2C12 according to [29] were cultured on conventional Petri dishes (BD Falcon) at 37°C and 5% CO_2_ in DMEM GlutaMAX (Gibco) supplemented with 10% heat-inactivated foetal bovine serum (FBS, EuroClone), penicillin (100 IU/mL, Gibco), and streptomycin (100 mg/mL, Gibco). All the experiments were conducted for 10 days starting from a cellular confluence of 50%, to obtain a high degree of differentiation, and cells were divided into two experimental groups: the control group cultured in growth medium and the treated group cultured in growth medium with the addition of TMPyP4 (Sigma-Aldrich) to a final concentration of 12 *μ*M. Medium was changed every day as well as fresh addition of TMPyP4.

### 2.2. Cell Proliferation and Viability

A six-well multiwell plate containing 1.5 × 10^4^ C2C12 cells for each well was prepared for every experimental time point (1, 2, and 5 days) and every experimental condition (ctrl, 6, 12, 25, and 50 *μ*M of TMPyP4). The medium was changed every day. Cellular proliferation was evaluated by counting the cells of every well for each multiwell plate at the 1st-, 2nd-, and 5th-day time points. For cellular viability, cells of the last time point (5 days) were stained with 0.4% Trypan Blue Solution (ThermoFisher), and the percentage of live cells on total cell number was taken as the survival percentage.

### 2.3. Immunofluorescence Analysis

Cells were fixed with 2% PFA in PBS for 10 minutes at 4°C and processed for immunofluorescence analysis as previously described [30]. Briefly, samples were washed with PBS and blocked with 10% goat serum in PBS for 1 h at room temperature (RT). Subsequently, cells were incubated with the primary antibody anti-myosin heavy chain (MF20, mouse monoclonal, DHSB, diluted 1 : 2) for 1 h, followed by incubation with Alexa Fluor 488-conjugated goat anti-mouse IgG (H+L) (Thermo Fisher Scientific, diluted 1 : 200) for 1 h. Finally, nuclei were stained with 300 nM DAPI (Thermo Fisher Scientific) for 10 min. Specimens were viewed under a Nikon TE 2000 epifluorescence microscope equipped with a Photometrics CoolSNAP MYO CCD camera.

### 2.4. Immunoblotting Analysis

Cells from both the control and treated groups were trypsinized, suspended in PBS, and immediately centrifuged at 200*g* for 5 min. Then pellets were suspended in RIPA buffer (20 mM Tris/HCl, pH 7.4, 5 mM EDTA, 0.1% SDS, 1% NP40, 1% NaDOC, and Roche protease inhibitor cocktail). Homogenates were centrifuged at 12.000*g* for 10 min at 4°C to discard nuclei and cellular debris. Protein concentration was determined with bicinchoninic acid (BCA) protein assay (Pierce) using bovine serum albumin (BSA) as the standard. Total homogenates were separated by sodium dodecyl sulfate-polyacrylamide gel electrophoresis (SDS-PAGE) with a concentration opportunely chosen on the basis of molecular weight of the proteins analysed. For Western blot analysis, proteins were transferred onto Immobilon membranes (Amersham), saturated with 5% nonfat dry milk (Bio-Rad) in 0.1% Tween-20 (Sigma) PBS (blocking solution) and hybridized with 1 : 5 MF20 mouse monoclonal antibody (DHSB) or with 1 : 5000 anti-myogenin mouse monoclonal (F5D, SCBT) or with 1 : 5000 anti-vinculin rabbit polyclonal (ab73412, Abcam) for 1 h at RT. The filters were washed three times (15 min each at RT) with wash solution (0.1% Tween-20 in PBS) and then reacted with anti-mouse or anti-rabbit secondary antibody conjugated with 1 : 3000 horseradish peroxidase IgG (Bio-Rad) for 1 h at RT, washed three times, and finally visualized with ECL (Amersham). Optical density (OD) was calculated with ImageJ software.

### 2.5. PCR and Quantitative Real-Time PCR (qRT-PCR) Analysis

RNA was extracted from dishes using TRIzol (Thermo Fisher Scientific), then 2 *μ*g of RNA both for control and treated samples was retrotranscribed using the high-capacity cDNA reverse transcription kit (Thermo Fisher Scientific), according to the manufacturers' instructions. Glyceraldehyde 3-phosphate dehydrogenase (GAPDH) was selected as endogenous control after verifying its stable expression. For PCR end point analysis, the PCR end point GoTaq® Green Master Mix (Promega) was used to amplify and visualize by agarose gel electrophoresis and ethidium bromide staining samples of cDNA obtained through the following pairs of primers:

- MHC (230): Fw: GCGTAGAGAGCGTGTCCAA; Rv: TTGGGTAAAGGCCTGCTTCGT
- Myog (395): Fw: GAGGAAGTCTGTGTCGGTGG; Rv: CCACGATGGACGTAAGGGAG
- GAPDH (263): Fw: CCCTTAAGAGGGATGCTGCC; Rv: ACTGTGCCGTTGAATTTGCC

Quantitative RT-PCR analysis was performed with a real-time PCR thermocycler (LightCycler®, Roche). Each cDNA sample was amplified in triplicate using KAPA SYBR® FAST qPCR kit Master Mix (Kapa Biosystems). Relative mRNA levels were calculated by the delta delta CT method [31, 32]. Primer specificity was confirmed by melting curve analysis.

Primers used are listed below:

- GAPDH (109): Fw: CGACTTCAACAGCAACTC; Rv: GTAGCCGTATTCATTGTCAT
- MyoD1 (82): Fw: GGAAGGGAAGAGCAGAAG; Rv: AAGGACTACAACAACAACAAC
- Mrf4 (153): Fw: CCTTTCCACCTAATCATT; Rv: CGTTCCGATATAATAATAAGTAT
- Myf5 (95): Fw: AGCATCTACTGTCCTGAT; Rv: GTGATCCGATCCACAATG
- MHC (88): Fw: ACATATCAGAGTGAGGAG; Rv: TCTTGTAGGACTTGACTT
- Myogenin (88): Fw: ATTCACATAAGGCTAACAC; Rv: CATCTTTCTCTCCTCAGA
- Myomaker (94): Fw: GTTGCTTCACTCTGTTCA; Rv: TATTTACTGGTCTAGGGTTCT

### 2.6. Statistical Analysis

All experiments were performed in biological and technical triplicate (*n* = 9). Data were analysed using GraphPad Prism 7, and values were expressed as means ± standard error (SEM). Statistical significance was tested using Student's *t*-test. A probability of less than 5% (*P* < 0.05) was considered to be statistically significant.

### 2.7. Localization of Tetraplex in MyoD1-Related Genes and Conservation Analysis

**Figure 1:**
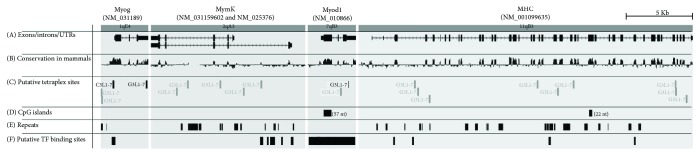
Multiregion view of the Myog, Mymk, Myod1, and MHC genomic regions. (A) Coding exons are represented by blocks connected by horizontal lines representing introns. The 5′ and 3′ untranslated regions (UTRs) are displayed as thinner blocks on the leading and trailing ends of the aligning regions. Arrowheads on the connecting intron lines indicate the direction of transcription. Mymk has multiple representations because two alternative splicings are known (NM_001159602 and NM_025376). (B) Sequence conservation of the considered region in mammals: CDS are the higher conserved regions, because of the pressure toward the maintenance of the codified peptides. Also, the UTR regions are largely conserved, supposedly for functional constraints. (C) Putative tetraplex sites, bioinformatically predicted on the forward or reverse strands (G3L1–7 and C3L1–7 labels, resp.): those in black have highly conserved sequence in mammals, that is, signal of both a conservative pressure against the divergence that leads to speciation and a function shared among all mammals. (D) CpG island site. False-positive prediction of tetraplex sites may be due to high concentration of G in these islands, but this is not the case of the four considered genes. (E) Repeats. Also, repeats with high percentage in GC may lead to false-positive tetraplex prediction, but this is not the case for the three conserved tetraplexes (black boxes in (C)). (F) Putative transcription factor-binding sites. The presence of both a tetraplex and a transcription factor-binding site in close proximity, as well as in the same sequence, strongly suggests that the two sites may collaborate to modulate the transcription factor effect on the genes.

Four regions were extracted from the *Mus musculus* genome draft (i.e., GRCm38/mm10, Dec. 2011 version). The genomic regions selected were those coding for the longest available mRNA of four murine genes representative for muscle differentiation: Myog (NM_031189.2), Mymk (NM_025376.3), MyoD1 (NM_010866.3), and MHC (NM_001099635.1). Each region was further extended to include 1 Kb of the genomic sequence upstream to the 5′ untranslated region (resp.: chr1:134,289,004-134,292,548; chr2:27,061,636-27,073,161; chr7:46,375,474-46,379,092; and chr11:67,077,300-67,102,291). Tetraplex searches were performed by sequence submission to the TetraplexFinder site (http://quadbase.igib.res.in/TetraPlexFinder). The results were graphically presented in the related genomic context taking advantage of the UCSC genome browser (http://genome-euro.ucsc.edu/cgi-bin/hgTracks?db=mm10) that allowed also the tetraplex sequence-conservation analysis. A confirmation test was performed searching for the four genomic selected sequences in the NCBI databases by blast algorithm (https://blast.ncbi.nlm.nih.gov/Blast.cgi), looking for conservation of each putative tetraplex site in the query-anchored alignment-view format of the BLASTN results (Figure 1).

### 2.8. Tetraplex Conservation in Murine MyoD1 Promoter

The MyoD1-analysed region was further extended to include the whole murine promoter, reaching the total size of ~8 Kb (chr7:46,370,861-46,379,092). The putative murine promoter was identified by similarity with the human version that was known to include a distal regulative region (DRR) and proximal regulative regions (PRR), both upstream to the 5′UTR of the human MYOD1 gene (NM_002478.4, chr11:17,719,563-17,722,131 on GRCh38/hg38 human genome draft) [33]. MyoD1 tetraplex sites (hypothesized first by Yafe and colleagues [23]) were searched, and conservation analysis was performed by the same TetraplexFinder/UCSC genome browser/BLASTN approach described in the previous paragraph.

## 3. Results

### 3.1. TMPyP4 Exposure Affects C2C12 Myogenic Differentiation

**Figure 2:**
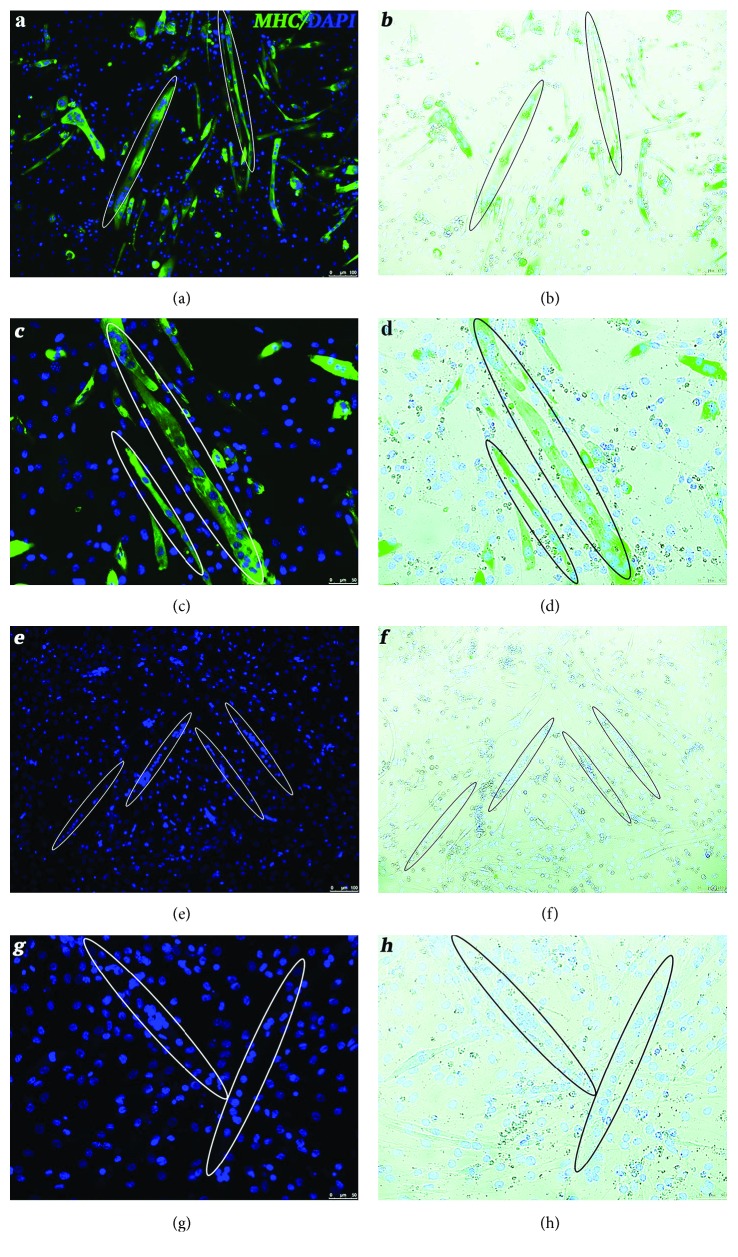
Immunofluorescence against MHC (green) on differentiating C2C12 cell culture untreated or treated with 12 mM of TMPyP4. (a–d) Untreated myoblast. In (a) 10x and (c) 20x, nuclei were counterstained by DAPI (blue). In (b) 10x and (d) 20x, immunofluorescence was superimposed on phase-contrast image. (e–h) TMPyP4-exposed differentiating C2C12 cells. In (e) 10x and (g) 20x, immunofluorescence was superimposed on phase-contrast image and nuclei were labelled by DAPI (blue). In (f) 10x and (h) 20x, no MHC presence was detected. Elliptical shapes highlighted myotube and elongated structure resembling a myotube not expressing MHC upon C2C12 cell TMPyP4 exposure.

We studied the effect of TMPyP4 on MyoD1 and downstream-regulated genes during myogenic differentiation by analysing the immortalized mouse myoblast cell line C2C12, which is able to form contractile myotubes and express specific muscle proteins during myogenic differentiation. In order to select the optimal concentration affecting MyoD1 and downstream target genes, different TMPyP4 concentrations have been tested, ranging from 6 to 50 *μ*M, on differentiating myoblasts. Cell survival, proliferation, and myogenic expression have been analysed revealing 12 *μ*M being the lowest concentration blocking MyoD1 and myogenin expression (Supplementary Figure 1) while leaving unaltered cell viability and proliferation (Supplementary Figure 2). Hence, we employed 12 *μ*M TMPyP4 treatment to study tetraplex influence on C2C12 during muscle differentiation (Figure 2). Porphyrin-exposed myoblast (after 10 days of culture) showed a normal behaviour reaching full confluence and syncytia formation with an elongated structured closing resembling myotubes (Figures 2(f) and 2(h)). Nevertheless, immunofluorescence against MHC revealed a full lack of MHC expression (Figures 2(e) and 2(g)). On the other side, the untreated control displayed muscle differentiation and complete myotube formation with multinucleated syncytia expressing MHC (Figures 2(a)–2(d)).

### 3.2. TMPyP4 Blocks MyoD1 Transcription

**Figure 3:**
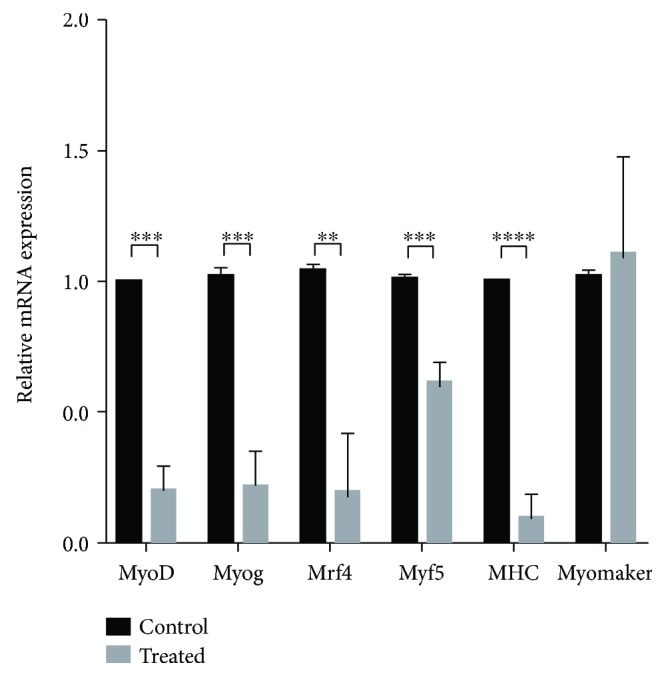
Gene transcription levels of Myod, Myog, MHC, and Mymk analysed by qRT-PCR (CTRL = controls; 12 *μ*M TMPyP4 = porphyrin-treated samples). Values of Myog, MHC, and Mymk normalized with respect to vinculin, in untreated and treated cells. Differences between treated cells (grey bars) and control cells (black bars) are highly significant for all genes, except for Mymk (^∗∗^ *p* < 0.01; ^∗∗∗^ *p* < 0.001; ^∗∗∗∗^ *p* < 0.0001).

Since the presence of tetraplex in the MyoD1 promoter is not only putative but also proved in *in vitro* analysis [22], we verified at the molecular level the effect of TMPyP4 on MyoD1 expression. Thus, mRNA transcription levels of treated and untreated C2C12 cells have been analysed. Myoblasts after 10 days of culture, as already described previously, underwent cellular fusion and syncytia formation as shown in Figure 2. MyoD1 transcript comparison in treated and untreated cells revealed TMPyP4 effect on the MyoD1 promoter region, inhibiting RNA transcription level. Quantitative RT-PCR (qRT-PCR) analysis revealed the remarkable inhibition (4-fold) of the MyoD1 transcription level upon TMPyP4 exposure compared with untreated control (Figure 3).

### 3.3. TMPyP4 Effect on Analysed Myogenic Genes

qRT-PCR performed on C2C12 untreated or treated with TMPyP4 (12 *μ*M) revealed, besides the inhibition of the MyoD1 RNA level, the downregulation of its downstream target genes Myog, MRF4, Myf5, and MHC transcription (Figure 3). TMPyP4 exposure affects MyoD1, Myog, and downstream myogenic genes, while leaving unaffected myomaker (Mymk), the gene driving cell fusion [34], normally transcribed in both treated and untreated cells (Figure 3).

**Figure 4:**
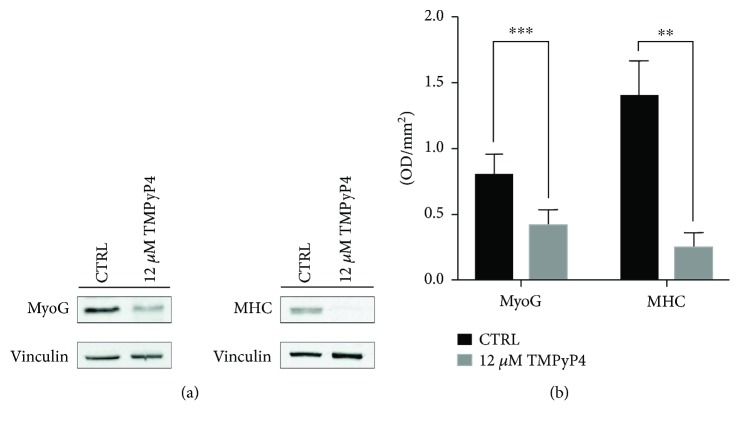
Western blot analysis for Myog and MHC protein expressions in differentiating C2C12-treated and untreated cells. (a) Representative Western blot filters labelled with antibodies against Myog, MHC, and vinculin as the loading control. (b) Optical densitometry analysis for quantitative evaluation of Myog and MHC expression normalized with vinculin. The differences between treated cell (grey bars) and control cells (black bars) are strongly significant both for MyoG and MHC protein expression (^∗∗^ *p* < 0.01; ^∗∗∗^ *p* < 0.001).

In order to verify whether TMPyP4 exposure effect on RNA transcription levels reflects impairing at the protein level too, Western blot analysis has been performed on C2C12 cells cultured for 10 days with or without tetraplex stabilizing porphyrin. Performed Western blot revealed a very significant different level of protein expression in treated and untreated cells (Figure 4), with a remarkable decrease of Myog and MHC proteins in exposed samples. The densitometric analysis revealed a decrease of 2-fold for Myog and 5-fold for MHC after vinculin normalization as the loading control (Figure 4).

### 3.4. Localization of Conserved Tetraplexes in MyoD1-Related Genes

The G-quartet investigation in four selected regions of the murine Myog, Mymk, MyoD1, and MHC genes identified 20 putative tetraplex sites (Figure 1). Most of the identified tetraplexes were found to be not conserved in other genomes of the Rodentia family, but 3 located in the Myog and MyoD1 regions are conserved (Figure 1). These three putative tetraplex sites are highly conserved in mammals and show signs of conservation also in species phylogenetically more distant from mice (see Supplementary Figure 3). It is worth to be noted that in these three cases, the highly conserved regions are not restricted to just the three tetraplexes but are embedded in wider highly conserved regions (Figure 1).

### 3.5. MyoD1 Promoter Conservation

The in silico analysis of the whole MyoD1 promoter region (~5 Kb) reports a nonhomogeneous conservation in mammals of the different parts of the promoter itself [33]. The most conserved portions are the distal regulative region (DRR) and proximal regulative regions (PRR), both upstream to the transcribed MyoD1 region. In particular, PRR shows a higher level of conservation than the other regulative region (Supplementary Figure 3). Including the MyoD1 promoter region in tetraplex detection analysis, one more putative site was identified, but its level of conservation in other genomes remains controversial because of its location to a simple repeat sequence (Supplementary Figure 4).

## 4. Discussion

The MyoD1 activity *in vivo* and *in vitro* on mammalian cells was studied in the last 30 years [33]. The protein has a specific DNA-binding domain, thus acting as a transcription factor of genes involved in myogenesis, interacting with other genes in a redundant manner, making nonlethal the null mutation in mice. The transcription factor DNA-binding activity depends on the accessibility of DNA and its spatial 3D arrangement. The porphyrin TMPyP4 binds DNA tetraplex sites stabilizing these structures [21, 35]. Our aim was to verify the activity of the tetraplex structure situated in the MyoD1 promoter region during myogenic differentiation process. We investigated the *in vitro* effect of TMPyP4 (12 *μ*M) during cell differentiation of the murine myoblast C2C12 cell line. Cells exposed to the porphyrin compound showed cell survival, proliferation, and fusion forming polynucleated syncytia, devoid of MHC expression. This effect, observed using immunofluorescence approaches, was further confirmed by both qRT-PCR and Western blot analysis. The qRT-PCR experiments assessed that MyoD1, Myog, MRF4, Myf5, and MHC are inhibited by the porphyrin effect, while leaving unperturbed Mymk. Furthermore, Western blot confirmed that also at the protein level, tetraplex stabilizing porphyrin impairs Myog and MHC expression. This effect agrees with the previous observation that in *Danio rerio* embryos, the Mymk activity is sufficient for myogenic cell fusion forming syncytia, despite the absence of MyoD1 activation essential for complete myotube differentiation [36]. Moreover, the in silico analysis on muscle-related genes showed only three highly conserved putative tetraplex sites. Since two of these tetraplexes were found in Myog promoter, it cannot be excluded that the TMPyP4 inhibition effect on MyoD1 occurs in a similar way also in myogenin.

## 5. Conclusions

To date, the relationship between the mechanism and regulation of myoblast fusion and muscular differentiation is still unclear, and it has been difficult to find evidences that these two processes can be uncoupled [34]. Our results on mammalian cells (*Mus musculus*) show the existence of two independent pathways for muscle differentiation and syncytia shaping, the latter being not influenced by the TMPyP4/tetraplex interaction. Our analyses suggest that at least one tetraplex is present in MyoD1 and two in Myog regulatory regions, with a conservation level that suggests a relevant functional activity of the three sites. Vice versa, no conserved tetraplex was found in the MHC gene. The tetraplex conservation could suggest a hierarchy among MyoD1 and Myog, which have these regulatory structures, and MHC, whose expression could be controlled by the first two genes. Thus, the presented work confirms previous work regarding the presence of G-quartet-derived tetraplex on the MyoD1 promoter sequence, further assuming the other two putative tetraplex regions on the myogenin sequence as well. Moreover, for the first time, we demonstrated the possibility to decouple myogenic differentiation and cell fusion inhibiting MyoD1 activity, exploiting porphyrin activity, and stabilizing the tetraplex region while leaving unaffected the Mymk fusion gene.

## Supplementary Materials

Supplementary Materials Supplementary Figure 1: standard PCR analysis for testing influence of TMPyP4 concentration on Myod1 and Myog expression. The image presents two different gels concerning Myod and Myog expression and GAPDH as internal control for differentiating C2C12 myoblasts untreated (CTRL) or treated with different tetraplex-stabilizing porphyrin concentration. Supplementary Figure 2: C2C12 proliferation and viability analyses upon TMPyP4 different concentration exposure. (a) Cell proliferation analysed over 5 days on treated or untreated (CTRL) differentiating myoblast. (b) Cell viability analysed in C2C12 cells exposed for 5 days to different TMPyP4 concentrations or not exposed as control (CTRL). Supplementary Figure 3: in-depth analysis of the G-quartet conservation in the three tetraplexes highly conserved in mammals (Figure 1): (a) C3L1–7 of Myod1; (b) C3L1–7 of Myog; (c) G3L1–7 of Myog. Supplementary Figure 4: search for tetraplex structures in the upstream region of the Myod1 gene that includes the whole Myod1 promoter (DRR: distal regulatory region; PRR: proximal regulatory region). No other tetraplex sequences are evidenced in the conserved sequences, confirming as the most probable candidate the highly conserved tetraplex at 3′UTR of the Myod1 transcript (see Figure 1).
