# Supplementary material for: Myoblast Myogenic Differentiation but Not Fusion Process Is Inhibited via MyoD Tetraplex Interaction
Source: Oxid Med Cell Longev. 2018 May 7;2018:7640272. doi: 10.1155/2018/7640272 (PMC5964432; doi:10.1155/2018/7640272)
Supplement: Supplementary Materials — Supplementary Figure 1: standard PCR analysis for testing influence of TMPyP4 concentration on Myod1 and Myog expression. The image presents two different gels concerning Myod and Myog expression and GAPDH as internal control for differentiating C2C12 myoblasts untreated (CTRL) or treated with different tetraplex-stabilizing porphyrin concentration. Supplementary Figure 2: C2C12 proliferation and viability analyses upon TMPyP4 different concentration exposure. (a) Cell proliferation analysed over 5 days on treated or untreated (CTRL) differentiating myoblast. (b) Cell viability analysed in C2C12 cells exposed for 5 days to different TMPyP4 concentrations or not exposed as control (CTRL). Supplementary Figure 3: in-depth analysis of the G-quartet conservation in the three tetraplexes highly conserved in mammals (Figure 1): (a) C3L1–7 of Myod1; (b) C3L1–7 of Myog; (c) G3L1–7 of Myog. Supplementary Figure 4: search for tetraplex structures in the upstream region of the Myod1 gene that includes the whole Myod1 promoter (DRR: distal regulatory region; PRR: proximal regulatory region). No other tetraplex sequences are evidenced in the conserved sequences, confirming as the most probable candidate the highly conserved tetraplex at 3′UTR of the Myod1 transcript (see Figure 1). [file 7640272.f1.pdf]

Supplementary materials

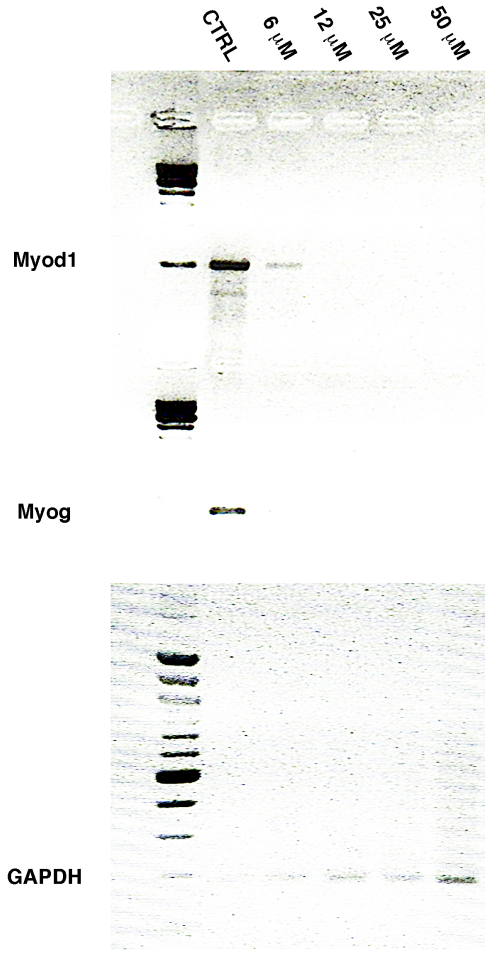

Supplementary Figure 1

Standard PCR analysis for testing influence of TMPyP4 concentration on Myod1 and Myog expression. The image present two different gels concerning Myod and Myog expression, and GAPDH as internal control for differentiating C2C12 myoblasts untreated (CTRL) or treated with different tetraplex stabilizing porphyrin concentration.

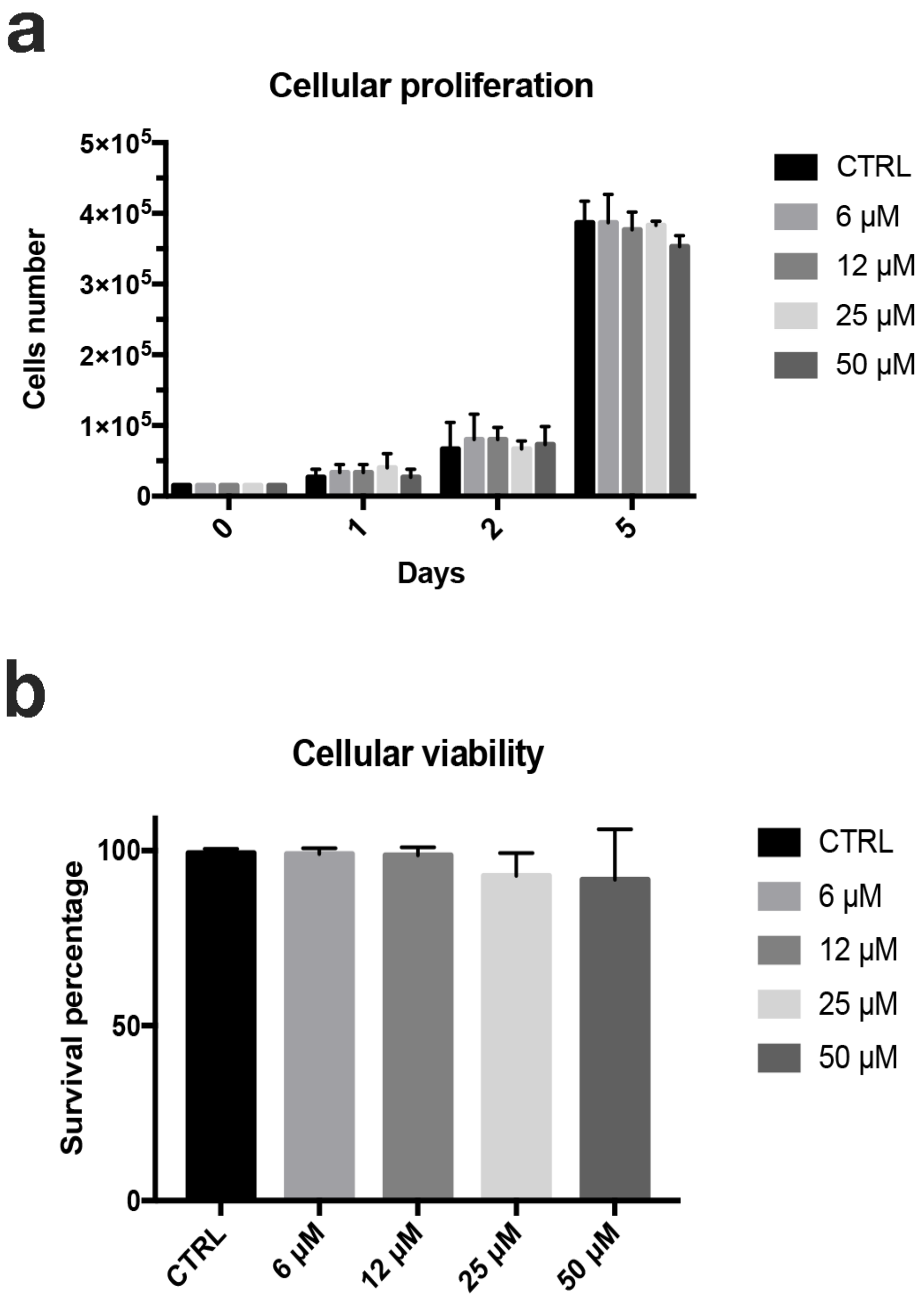

Supplementary Figure2
C2C12 proliferation and viability analyses upon TMPyP4 different concentrations exposure. (a) Cell proliferation analysed over 5 days on treated or untreated (CTRL) differentiating myoblast; (b) Cell viability analysed in C2C12 cells exposed for 5 days to different TMPyP4 concentration or not exposed as control (CTRL).

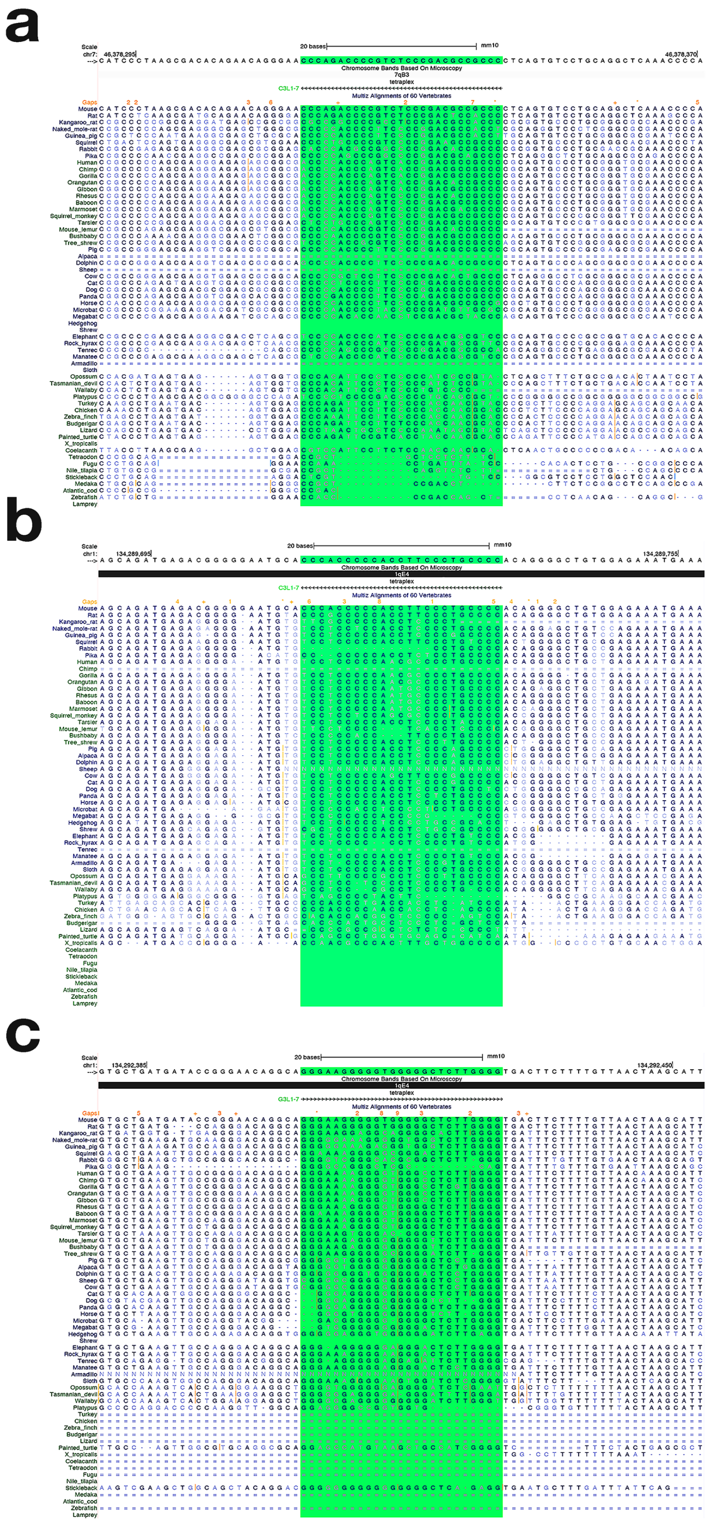

Supplementary Figure 3

In depth analysis of the G-quartets conservation in the three tetraplexes highly conserved in mammals (red boxes in figure 4): (a) C3L1-7 of Myod1; (b) C3L1-7 of Myog; (c) G3L1-7 of Myog.

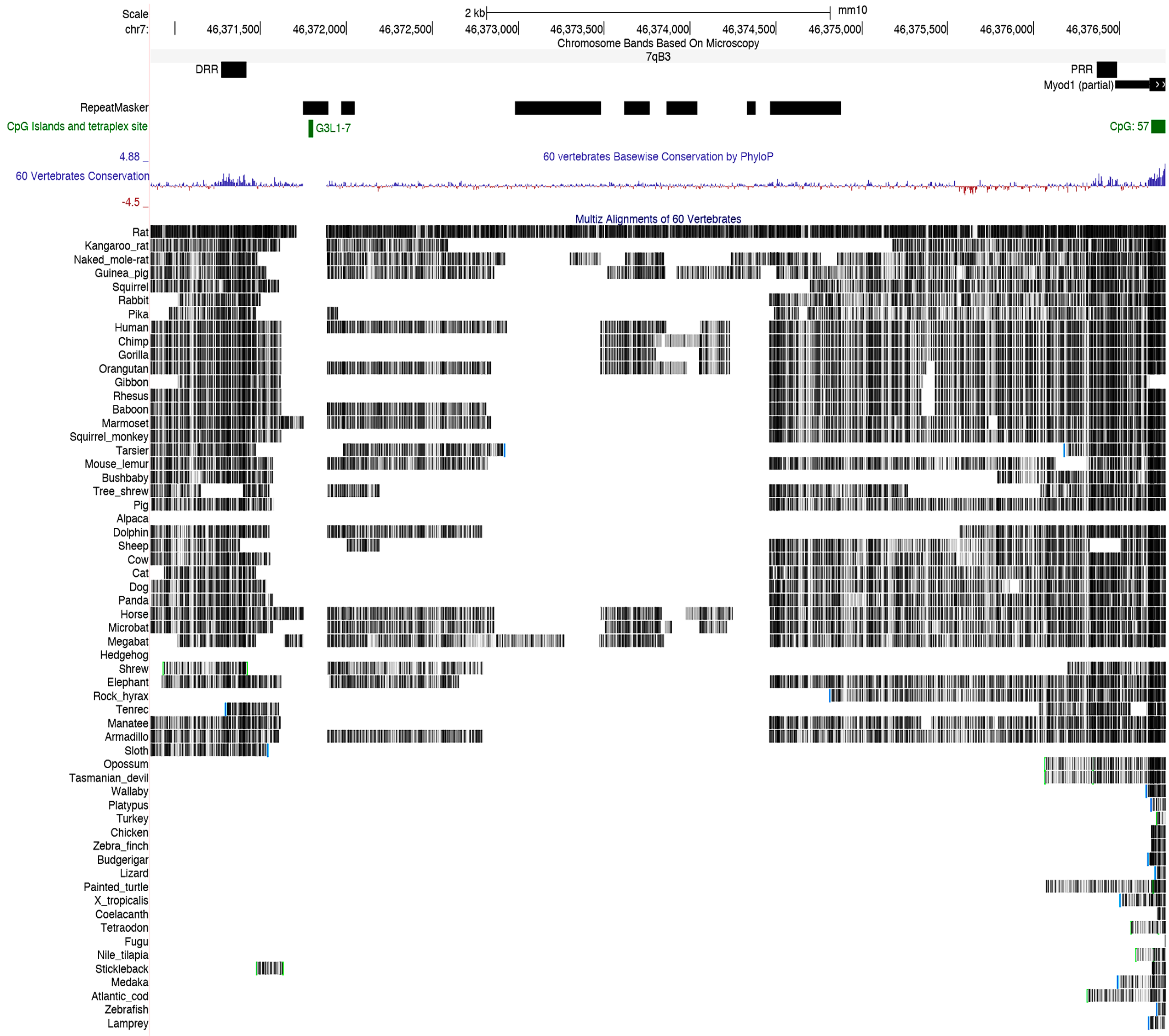

Supplementary Figure 4

Search for tetraplex structures in the upstream region of the Myod1 gene, that includes the whole Myod1 promoter (DRR: Distal Regulatory Region; PRR: Proximal Regulatory Region). No other tetraplex sequences are evidenced in the conserved sequences, confirming as the most probable candidate the highly conserved tetraplex at 3'UTR of Myod1 transcript (see figure 4).
